# Activity-Based Fluorescent Probes Based on Hemicyanine for Biomedical Sensing

**DOI:** 10.3390/molecules27227750

**Published:** 2022-11-10

**Authors:** Pan Luo, Min Wang, Wenguang Liu, Lin Liu, Peng Xu

**Affiliations:** Department of Joint Surgery, HongHui Hospital, Xi’an Jiaotong University, Xi’an 710054, China

**Keywords:** hemicyanine, fluorescent probe, imaging, sensing, analytical tool

## Abstract

In recent years, fluorescent probes, as an analytical tool that can target and rapidly detect analytes, have been increasingly used for applications related to medical treatment, detection, and bioimaging. Researchers are interested in hemicyanine-based fluorescent probes because of their high quantum yield, tunable spectrum characteristics, absorption and emission in the near-infrared (NIR) region, and good photo-stability. The development of these dyes and their derivatives as NIR fluorescent probes for biological applications has advanced significantly in the last ten years. This review introduces processes for making hemicyanine dyes and the methodology for creating functional activity-based fluorescent probes. A variety of hemicyanine-based probes have been systematically developed for the detection of small biomolecules in various illnesses. Finally, the potential drawbacks of hemicyanine-based functional probes, and the prospects for future research and translation into clinical medicine, are also discussed. This study is intended to provide strategies for the development and design of novel fluorescence probes.

## 1. Introduction

As a trace analysis and imaging tool, fluorescent probes have been widely used in environmental detection, medicine, and other fields [1,2]. Compared with general instrumental methods [1] (atomic absorption spectrometry, titration, and electrochemical methods), fluorescence analysis and its imaging techniques demonstrate various advantages under simulated physiological conditions, such as a low cost, simple operation, and high precision and sensitivity, and they can also be used for real-time detection and visualization in vivo [3,4,5,6,7,8,9,10].

Recently, various probes based on distinct fluorophores for the detection of analytes in biological systems have been created, such as fluorescein, coumarin, rhodamine, cyanine, and hemicyanine [11,12,13,14]. However, NIR fluorescent probes have gained a lot of interest due to their distinctive advantages, such as their deep tissue penetration capability and good signal-to-noise ratio, as well as sufficient sensitivity to greatly reduce the generation of false positive signals, especially in the fields of bioimaging and biomedicine [15,16,17,18,19,20,21,22]. The excitation and emission of hemicyanine fluorophores are not only in the near-infrared range, but they also have high fluorescence quantum yield and good biocompatibility [23,24]. These advantages have prompted many researchers to use hemicyanine as a template to study novel fluorescent probes [25].

Traditional cyanine dyes have low fluorescence quantum yields, which may be a limitation in biological imaging [26,27]. In particular, the highly conjugated cyanine dyes are not very stable, in terms of their chemical properties, and their structure is easily destroyed by strong oxidizing reagents [28,29]. Therefore, it is in the interest of researchers to transform and optimize such dyes, to improve their quantum yield and stability. Hemicyanine dyes with a donor-π-acceptor (D-π-A) motif were developed to be used as promising fluorophores for NIR bioimaging. Hemicyanine-based activatable NIR probes have been created for the detection of various analytes and the diagnosis of certain disorders, such as arthritis, neuropathy, and cancer [30,31,32]. The design strategies for these probes were mainly based on intramolecular charge transfer, which is mainly attributed to the structure of the D-π-A of hemicyanine [33]. Briefly, by combining a specific responsive group of a molecule with a modifiable moiety (usually an amino or hydroxyl group) of a hemicyanine, the intramolecular charge transfer (ICT) mechanism is disrupted, resulting in a low background fluorescence. However, when a specific molecule is present, the ICT mechanism is restored and a strong fluorescent signal is released, thereby achieving specific detection of the target molecule.

**Scheme 1:**
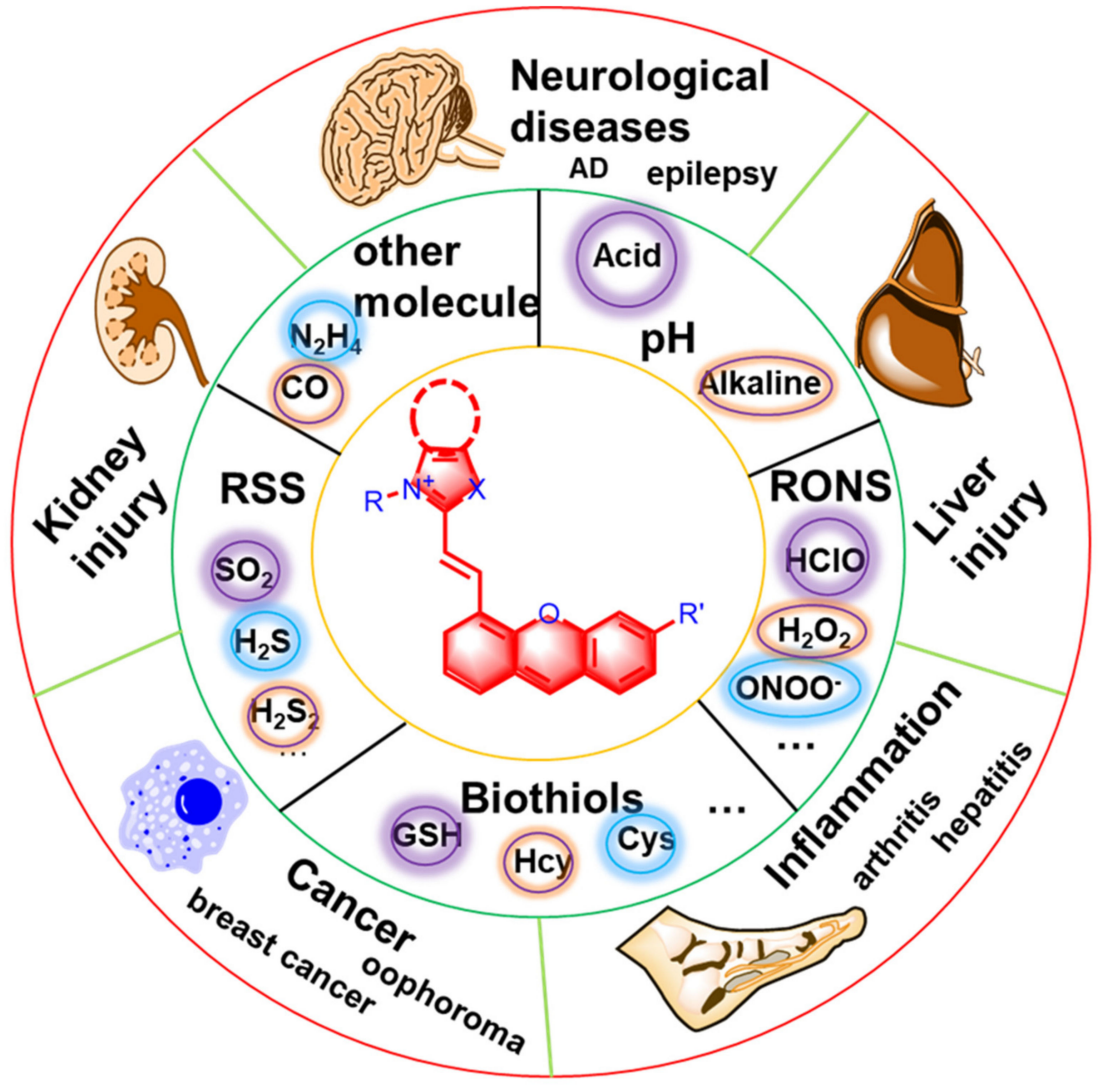
Overview of hemicyanine fluorescent probes for detecting small molecular markers of disease activity.

The dynamic redox homeostasis in the living body is inseparable from normal intracellular physiological processes [34,35]. However, once this balance is disrupted, oxidative stress will increase rapidly, potentially causing damage to linked biological factors, which can ultimately result in a variety of illnesses, such as cancer, inflammatory diseases, and neurological illnesses (Scheme 1) [36,37,38]. Reactive oxygen species (ROS), reactive nitrogen species (RNS), reactive sulfur species (RSS), biological thiols, enzymes, and others are among the tiny molecules found in living organisms that are engaged in a variety of physiological and pathological functions [39,40]. For instance, peroxynitrite (ONOO^−^) can interact with proteins, lipids, RNA, and other substances to cause irreparable damage, and thus indirectly or directly induce the occurrence of certain diseases due to its strong oxidative and nucleophilic properties [41,42].

The primary starting points of this study are the hemicyanine dye manufacturing processes and probe design methodologies. The probes based on hemicyanine for the detection of some biomolecules are systematically summarized. Finally, the current development status and future challenges of hemicyanine fluorescent probes in this field are discussed.

## 2. The Structure and Design Strategy of Hemicyanine Probes

**Figure 1:**
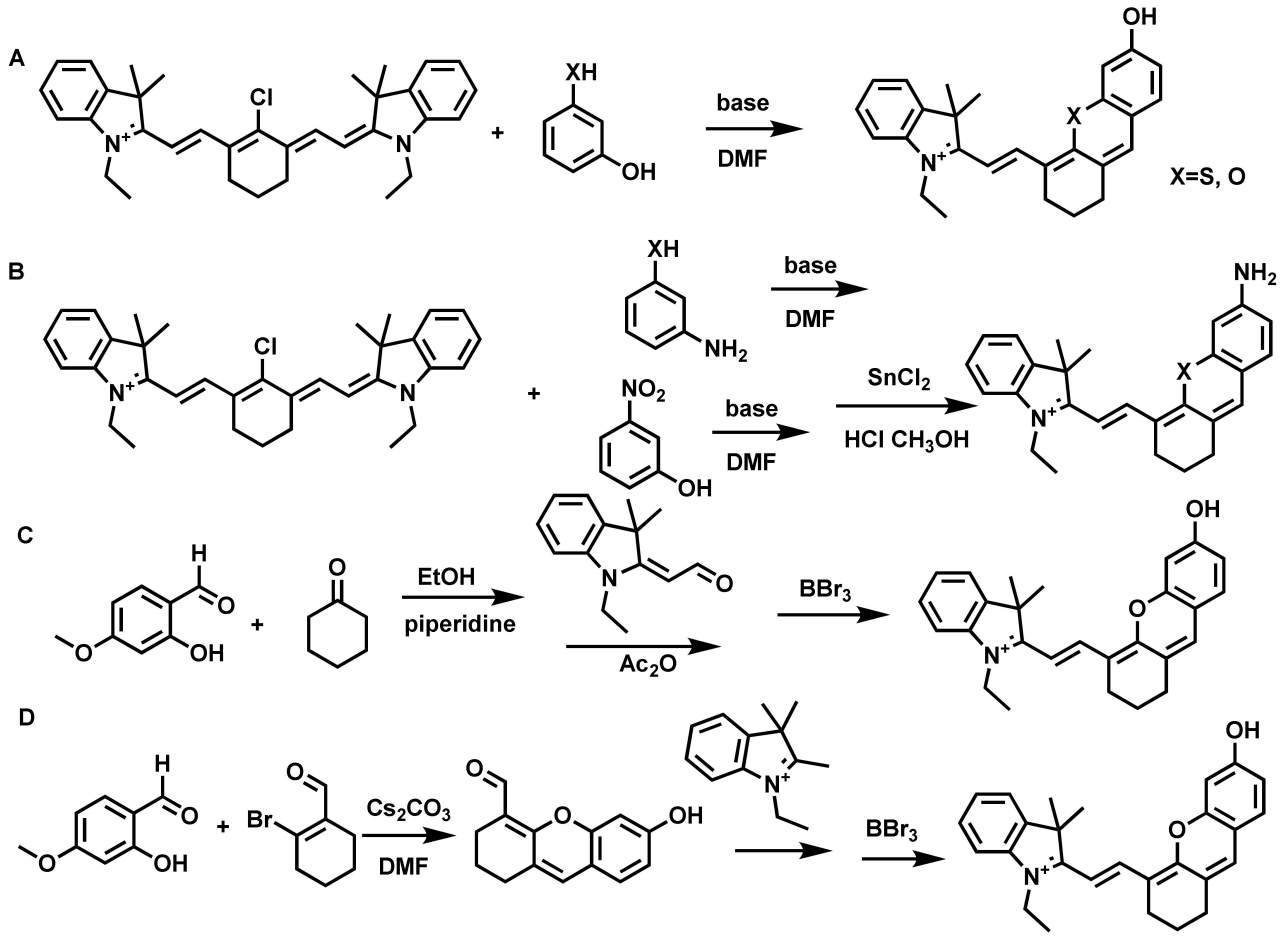
Synthetic method for preparing a hemicyanine scaffold. (**A**) Synthesis of hydroxylated hemicyanine scaffold. (**B**) Synthesis of amine-based hemicyanine scaffold. (**C**) Synthesis of improved hemicyanine scaffold. (**D**) Synthesis of the simplest hemicyanine scaffold.

Since the discovery of hemicyanine fluorophores, they have been widely used in biology, chemistry, and biomedicine. The methods for their synthesis are also a research hotspot. Several simple and representative synthesis methods are reviewed in this paper. Since the hemicyanine group and the cyanine group are structurally similar, researchers have previously prepared hemicyanine using cyanine. As shown in Figure 1, chloro-substituted cyanine dyes are converted into hemicyanine fluorophores under heating [43]. However, this method is relatively limited and not easy for the development of hemicyanine derivatives. In contrast, de novo synthesis methods facilitate the design of fluorescent derivatives of hemicyanine. Unfortunately, aldehyde derivatives are not easy to prepare and the yields are low. In 2015, Richard proposed a synthetic method that solved this problem and greatly promoted the development of hemicyanine dyes [44].

## 3. Progress of Hemicyanine-Based Fluorescent Probes

Effective fluorescent probes generally have the following properties. First, the probe has a low background signal, high quantum yield, and tunable optical properties. Second, the analyte can respond specifically, the response is rapid, and the detection limit is low. Since the biological system environment is complex and changeable, this requires probes with a high selectivity. Third, photostability is also an important aspect in the design of fluorescent probes. Finally, the probe should have good water solubility and biocompatibility, which lays a good foundation for its application in vivo.

**Figure 2:**
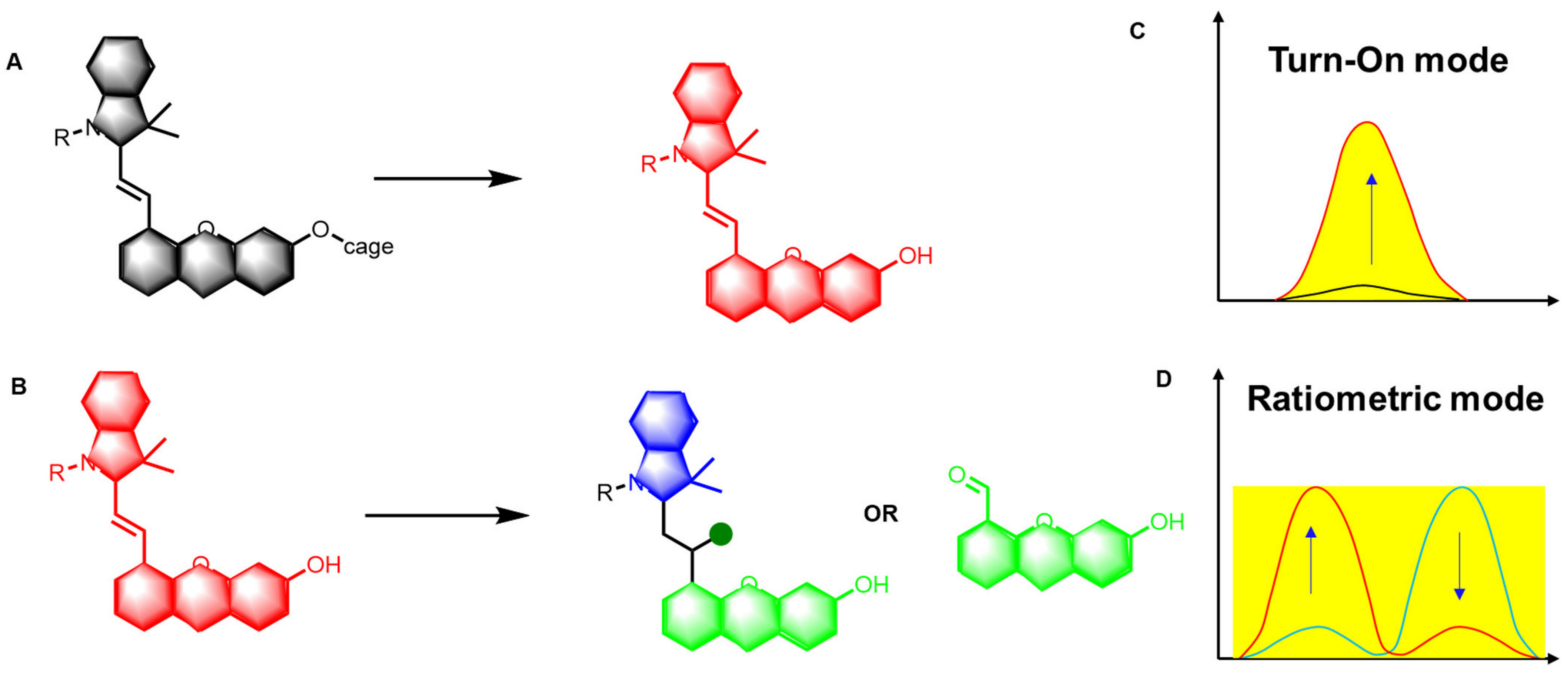
Design principles for activatable fluorescence probes. (**A**) Design principles of turn-on fluorescent probes. (**B**) Design principles of ratio-based fluorescent probes. (**C**) Image description of turn-on fluorescent probes. (**D**) Image description of ratio-based fluorescent probes.

The complex diversity of the synthesis and structure of hemicyanine makes it easy to incorporate functional units such as hydroxyl and amino groups. The responsive group of the probe is specifically cleaved when exposed to a specific biomarker, triggering an intramolecular charge transfer (ICT) process, which further generates a hydroxyl- or amino-containing hemicyanine-like fluorophore derivative, resulting in an increase in the fluorescence signal (Figure 2A) [45]. This ICT mechanism is mainly achieved by changing the electron-donating ability or interfering with the conjugation system of the hemicyanine. Additionally, the overall approach in the design of hemicyanine-based fluorescent probes is to destroy their large conjugated systems through nucleophilic-addition reactions or oxidative cleavage, using molecules such as ROS (such as hydrochloric acid (HClO)), RNS (such as ONOO^−^), and RSS (such as hydrogen sulfide (H_2_S)) (Figure 2B) [46]. Therefore, based on different design strategies, specific recognition of target molecules can be achieved, so as to achieve detection or diagnosis.

## 4. Progress in Hemicyanine-Based Fluorescent Probes

### 4.1. Detecting Protons Using Fluorescent Probes (H^+^)

Numerous physiological and pathological settings, including cell development, proliferation, and ion transport, depend heavily on pH. Under normal physiological conditions, the pH value in cells is generally controlled at 7.2–7.4 [47]. However, the pH value of different organelles is slightly different. For example, the pH value of lysosomes is approximately 4.5, whereas the pH value of mitochondria is slightly alkaline [48]. Abnormalities of pH may cause physiological disorders, which in turn may cause the development of a number of other disorders, including cancer and neurological diseases. Therefore, the detection of intracellular pH is crucial for understanding the function of cells and the diagnosis of certain diseases. In recent years, a number of fluorescent probes with hemicyanine as a parent have been created.

**Figure 3:**
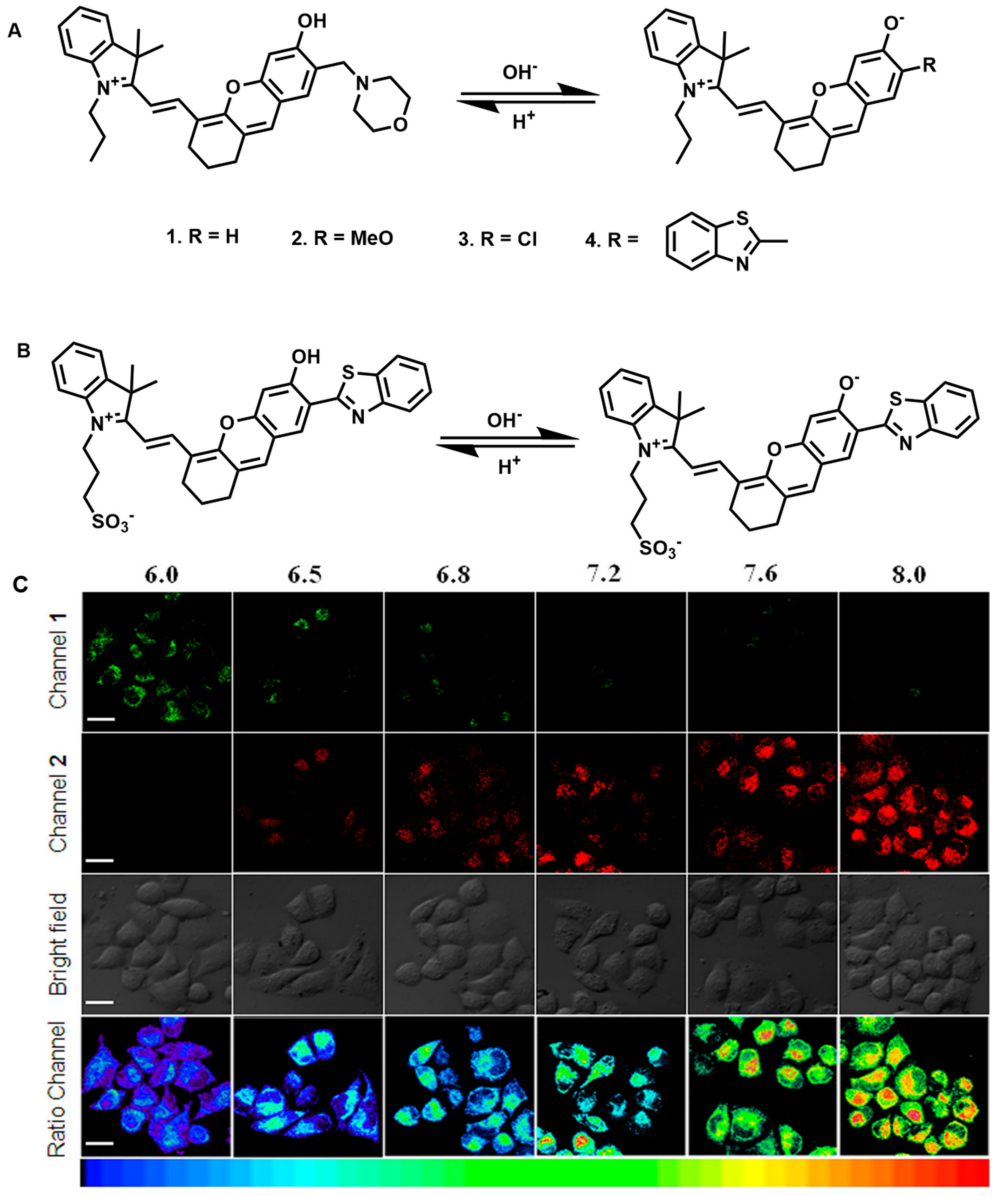
Responsive mechanism for pH and structures (**A**,**B**). (**C**) Fluorescent images in HeLa cells clamped at pH 6.0, 6.5, 6.8, 7.2, 7.6, and 8.0, respectively. Reproduced with permission from Ref. [49]. Copyright (2015) American Chemical Society.

pH-sensitive hemicyanine dyes have a phenolic hydroxyl group that can generate a ratiometric response to pH through a protonation/deprotonation mechanism. Yang and colleagues created several fluorescent probes using hemicyanine dyes for the detection of pH (Figure 3A) [49]. The Stokes shift of probe 4 was the largest, being in the pH range of 3.0–9.0. In addition, its emission wavelength was linearly related to pH in the range of 6.5 to 8.0 and was red-shifted from 672 to 750 nm. The probe was effectively used to measure the pH in vivo and in cells. Yu et al. improved the water solubility of their probe and extended its excitation and emission into the near-infrared region by introducing a sulfonic acid group (Figure 3B) [50]. Furthermore, this probe was used to detect acidosis caused by inflammation at the organism level.

**Figure 4:**
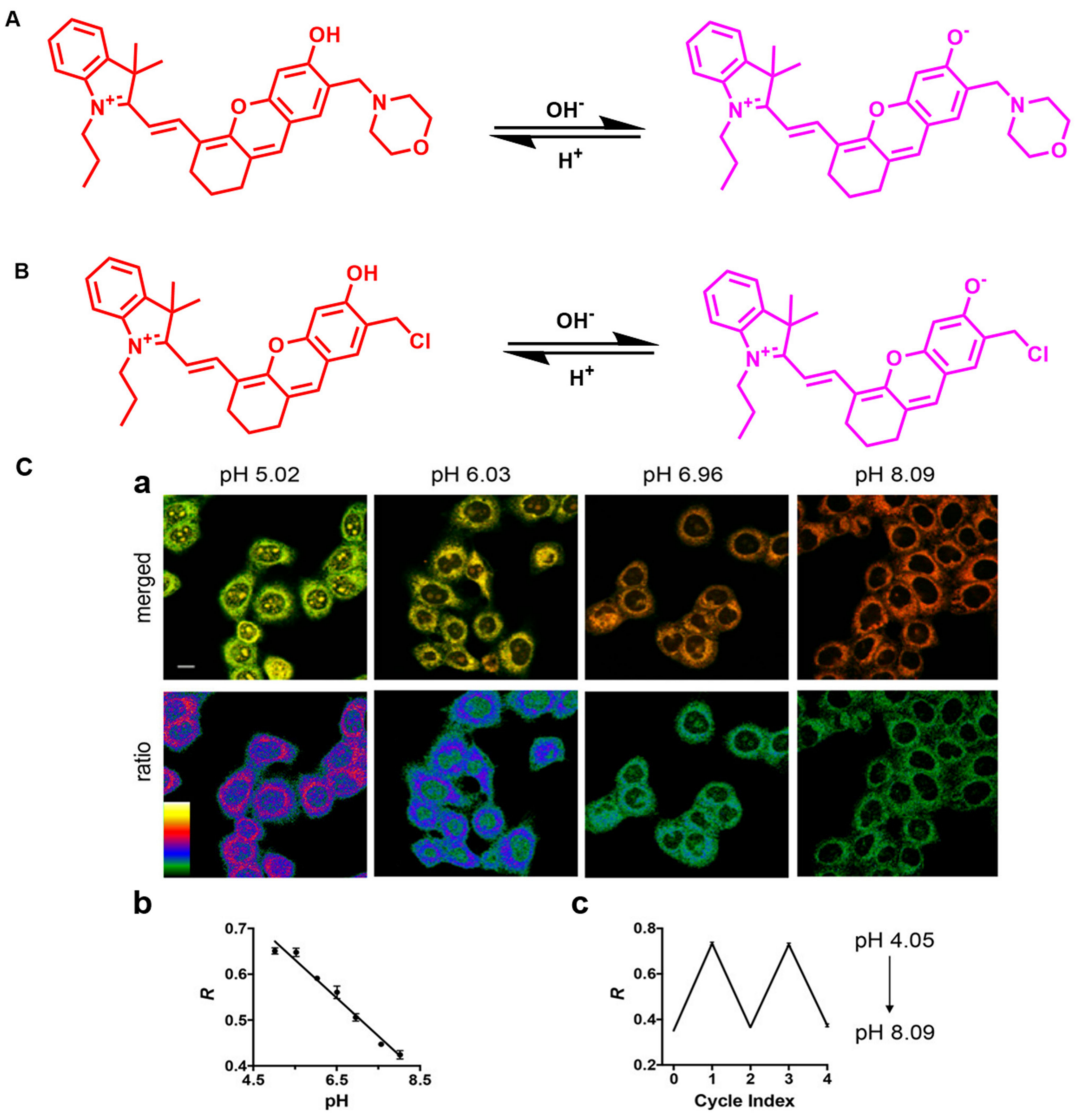
(**A**) Lysosome- and (**B**) mitochondrial-targeted pH fluorescent probes. (**C**) Calibration of intracellular pH in HeLa cells with HXPI-Cl. (**a**) Merged and ratio images of the green and red channels in different pH. (**b**) Linear relationship between the ratio value (R = Igreen/Ired) and pH in HeLa cells. (**c**) Reversibility of HXPI-Cl between pH 4.05 and pH 8.09 in HeLa cells. Reproduced with permission from Ref. [32]. Copyright (2019) American Chemical Society.

Since many physiological processes occur in organelles, Ma et al. introduced Morpholine, a typical lysosome-targeting moiety, to the hemicyanine backbone through modification (Figure 4A) [51]. This near-infrared probe can sensitively and reversibly detect changes in pH. In addition, in cell colocalization experiments, the Pearson coefficient was as high as 0.90 or more. The authors were the first to have studied the irreversible elevation of pH in lysosomes during heat shock. Subsequently, the research team substituted a benzyl chloride group for a hydroxyl group, in order to analyze the pH fluctuations in other organelles, allowing it to effectively accumulate near the mitochondria (Figure 4B) [32].

**Figure 5:**
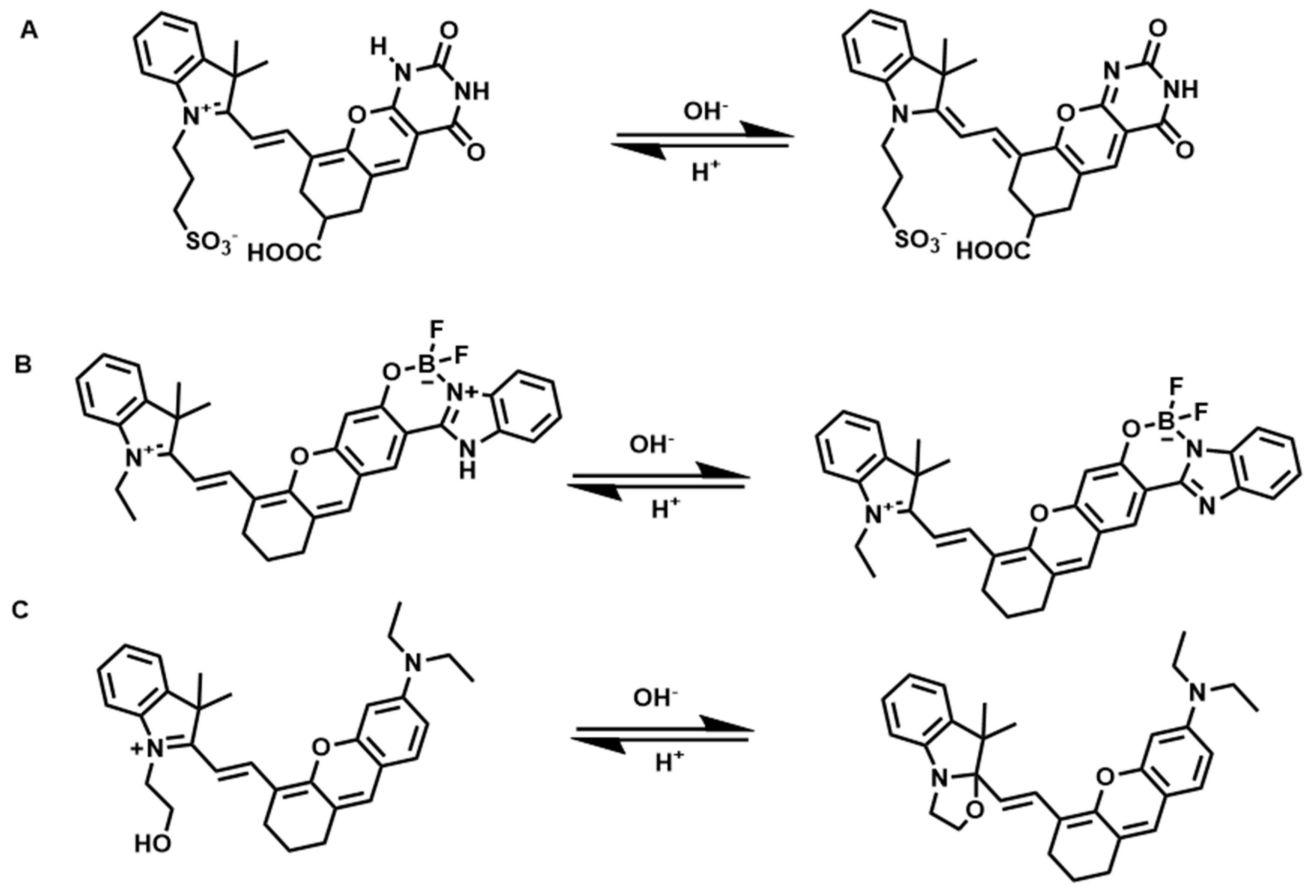
Responsive mechanisms for pH based on (**A**) uracil-containing hemicyanine dyes, (**B**) hemicyanine–boron fluoride conjugate and (**C**) oxazolidine switch in hemicyanine dyes.

Not only can hemicyanine dyes with phenolic hydroxyl groups be deprotonated, but free -NH can also change its ability to give and lose electrons by deprotonation, to achieve the effect of pH detection. Achilefu et al. developed an uracil-containing hemimethylcyanine dye for the detection of pH (Figure 5A). This mostly functioned through the deprotonation of uracil’s N-H bond [52]. A fluorescent probe consisting of a hemicyanine–boron fluoride conjugate for the measurement of pH (2.0–9.0) in lysosomes was created by Zhao et al. (2019) and successfully used to detect pH in cells and in vivo (Figure 5B) [53]. Liu et al. prepared near-infrared fluorescent probes by introducing an oxazolidine switch in hemicyanine dyes (Figure 5C) [54]. The probe exhibited a fast and sensitive response at pH values from 5.0 to 10.0. In addition, the probe had a high biocompatibility, high photostability, and low cytotoxicity, and was utilized to determine changes in mitochondrial pH.

### 4.2. Hydrogen Peroxide Fluorescent Probes

H_2_O_2_ is crucial to numerous physiological and pathological processes, as a second messenger of signal transduction and an important marker of oxidative stress. When the body’s level of H_2_O_2_ is aberrant, this will result in numerous physiological disturbances and the development of illnesses such as Alzheimer’s disease, diabetes, inflammation, and cancer. The important physiological functions of H_2_O_2_ make it important to design sensitive H_2_O_2_ detection methods and conduct in-depth studies [20,55,56,57,58,59].

**Figure 6:**
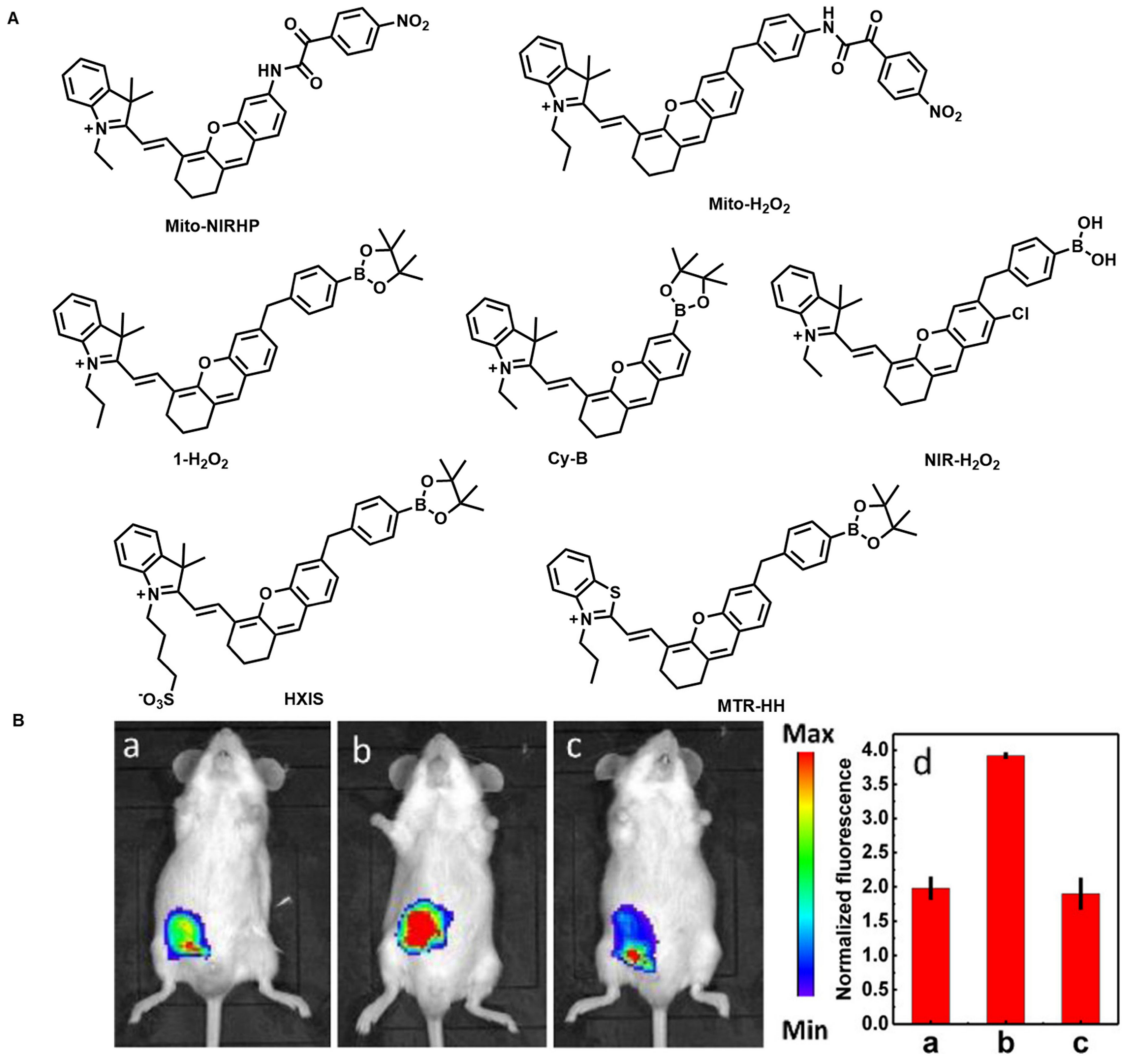
Chemical structures of fluorescent probes (**A**) for H_2_O_2_. (**B**) Fluorescence imaging (pseudocolor) of H_2_O_2_ in Kunming mice. (**a**) Only the Mito-NIRHP (20 μM, 100 μL) was injected as control. (**b**) Mouse pretreated with rotenone for 1 h and then injected with Mito-NIRHP. (**c**) Mouse successively injected with rotenone, NAC, and Mito-NIRHP. (**d**) Relative fluorescence intensities of (a)–(c). The excitation filter was 660 nm, and the emission filter was 710 nm. Reproduced with permission from Ref. [20]. Copyright (2016) American Chemical Society.

Tang et al. created a mitochondria-localized NIR fluorescence probe (Mito-NIRHP) (Figure 6). When H_2_O_2_ is present, the probe fluoresces intensely at 700 nm. Mito-NIRHP also demonstrated a good H_2_O_2_ specificity and sensitivity [20]. The probe was effectively used to detect H_2_O_2_ in living cells and in vivo, owing to its excellent biocompatibility and photophysical characteristics. A novel fluorescent probe called Mito-H_2_O_2_ was created and manufactured by Cao et al. using a similar concept (Figure 6). The detection limit for this probe was 186 nM. Mito-H_2_O_2_ has been successfully used to detect H_2_O_2_ in mouse models and potentially may be a reliable tool to study the relationship between H_2_O_2_ and related diseases [55]. As a known responsive group for H_2_O_2_, boronate esters show high specificity for H_2_O_2_. In 2016, Peng et al. introduced it into the hemicyanine skeleton and synthesized an NIR mitochondrial-targeted fluorescent probe, Cy-B (Figure 6) [56]. When H_2_O_2_ is present, the ICT mechanism is triggered and the fluorescence signal is rapidly enhanced. This probe was successfully applied in the study of autophagy. Subsequently, Zhang et al. modified it slightly by changing the boronate ester to a phenylboronic acid pinacol ester and developed the probe 1-H_2_O_2_ (Figure 6) [57]. This probe has high selectivity for some biological particles, such as amino acids and carbohydrates. When rotenone induction is present, the probe can track alterations in intracellular H_2_O_2_ concentrations. A comparable fluorescent probe with great sensitivity and a roughly 180-fold boost in fluorescence intensity was described by Lin et al. in the presence of H_2_O_2_. The probe had minimal cytotoxicity and worked well for detecting H_2_O_2_ in inflammatory model cells [43]. In 2022, a small-molecule fluorescent probe (HXIS) for H_2_O_2_ detection was disclosed by Zhang et al. (Figure 6) [58]. In the design of the probe, it was made more water-soluble with the addition of a sulfonic acid group. HXIS showed low cytotoxicity and good selectivity. This probe was the first to use a paper chip as a carrier to detect H_2_O_2_ using fluorescent signals, and it has great application potential in biological systems and for in vivo research. Feng et al. changed hemi-indocyanine and hemi-benzindocyanine to hemi-benzothiazolecyanine and created an NIR fluorescent probe, whose fluorescence intensity increased 284-fold when H_2_O_2_ was present (Figure 6) [59]. This is the strongest near-infrared fluorescent probe for H_2_O_2_ detection that has been documented.

### 4.3. Fluorescent Probes for the Detection of HClO

**Figure 7:**
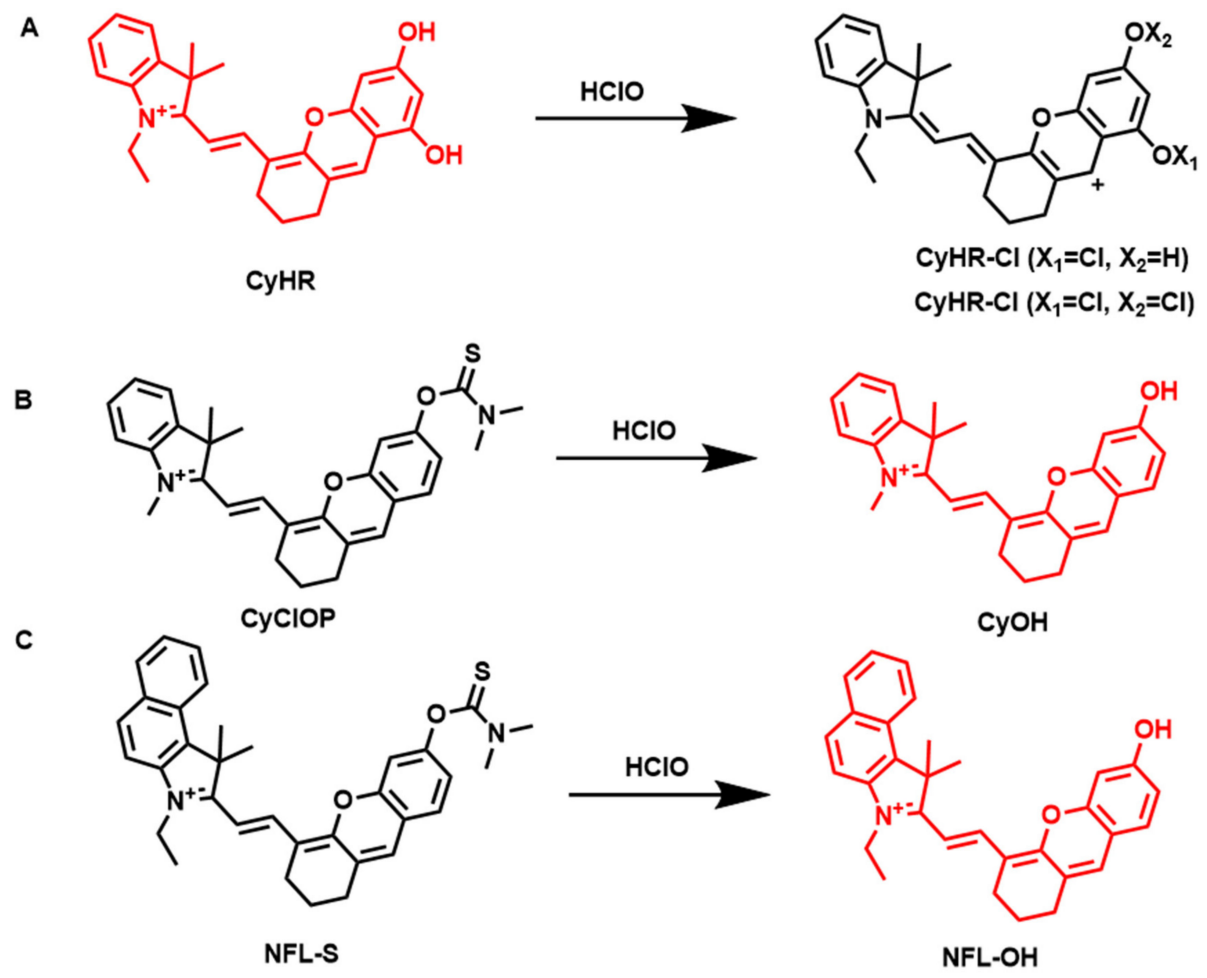
The responsive mechanisms and chemical structures (**A**) CyHR, (**B**) CyClOP and (**C**) NFL-S of the fluorescent probe for HClO.

An extraordinarily significant endogenous ROS, HClO, is involved in a number of physiological and pathological processes. Additionally, it is created by the heme enzyme myeloperoxidase (MPO) and the reactions of H_2_O_2_ and chloride ions (Cl-). Abnormal levels of it can lead to a range of diseases, such as atherosclerosis, liver cirrhosis, inflammation, and even cancer. However, a certain amount of HClO can also play a role in disinfection and resist the invasion of foreign species, such as bacteria, pathogens, etc. Hence, it is imperative to create a reliable method for determining the concentration of HClO in living organisms. The NIR fluorescent probe for HClO detection CyHR was created by Guo et al. in 2018 and primarily relies on the production of chlorine–oxygen (Cl-O) bonds to identify HClO (Figure 7A) [60]. Cl-O bonds had never previously been discovered in organic aromatic compounds until this investigation. The probe can respond quickly to HClO, requiring only 0.2 s, and the detection can be as low as 39.44 nM. Subsequently, a fluorescent probe (CyClOP) with great specificity for HClO was created by Lin et al. (Figure 7B). The responsive group of HClO employed in the probe is N-dimethylthiocarbamate, while the parent dye used in the probe is hemicyanine [61]. The probe can respond to HClO and reach a plateau within 5 s. Furthermore, CyClOP has low cytotoxicity and high photostability. Based on the above conditions, the probes were used to track the amounts of HClO in cells and in living organisms. In the same year, Liu et al. created the fluorescent NFL-S probe to detect HClO (Figure 7C) [62]. The probe was used to measure the level of HClO in mouse liver and arthritic tissues.

### 4.4. Fluorescent Probes for Superoxide Anion Detection (O_2_˙^−^)

**Figure 8:**
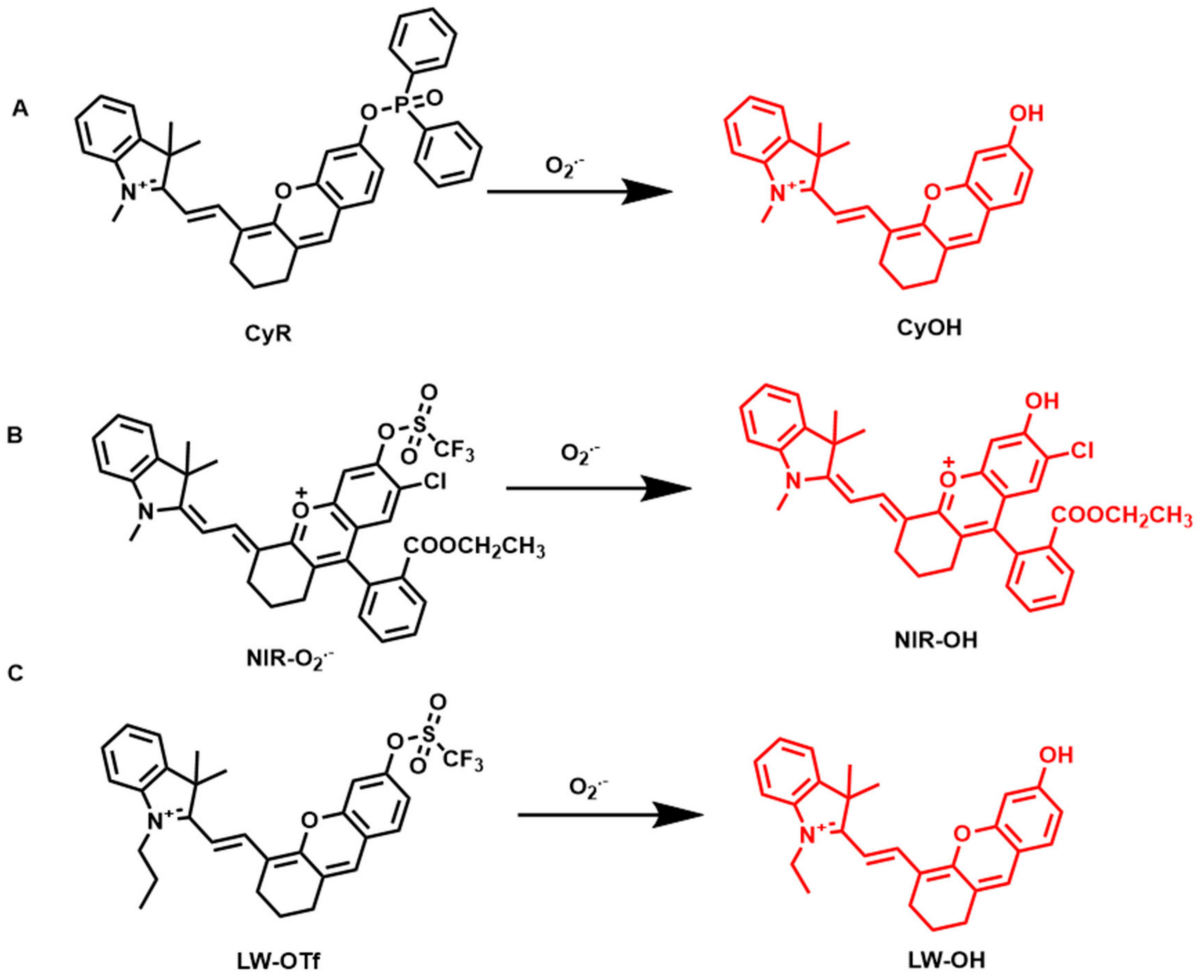
The chemical composition and response mechanisms (**A**) CyR, (**B**) NIR- O_2_˙^−^ and (**C**) LW-OTf of the fluorescence probe for O_2_˙^−^.

O_2_˙^−^ can be transformed into oxygen (O_2_) and hydrogen peroxide (H_2_O_2_) by manganese superoxide dismutase, causing extra oxidative stress. Nitric oxide (NO) produced by the body, oxygen, and O_2_˙^−^ can combine to form ONOO^−^, abnormal levels of which can cause a series of diseases, with liver-related diseases being of particular concern. In 2016, Zhang et al. developed a novel fluorescent probe CyR for the detection of O_2_˙^−^ (Figure 8A). Here, diphenylphosphonic acid was introduced as a recognition group for O_2_˙^−^, and exhibited a specificity response [63]. In the presence of O_2_˙^−^, the diphenylphosphonic acid group is deprotected, releasing the naked hydroxyl fluorophore, and the fluorescence signal is enhanced at 700 nm. Furthermore, CyR exhibits low cytotoxicity and good biocompatibility. Based on its good photophysical properties, it has been effectively used to detect oxygen in zebrafish, mice, and live cells. Zhang et al. subsequently created the mitochondria-targeting NIR fluorescence probe NIR-O_2_˙^−^. This probe is very effective for both in vitro and in vivo O_2_˙^−^ monitoring (Figure 8B) [64]. It has been used to successfully identify endogenously produced oxygen in living cells and tissues and to track changes in oxygen levels during drug-induced nephrotoxicity. In addition, the probe NIR-O_2_˙^−^ was used, for the first time, to elucidate the protective effect of L-carnitine (LC) against drug-induced nephrotoxicity. Therefore, this probe may be a potential chemical tool for exploring the role of O_2_˙^−^ in complex nephrotoxic disease systems. Subsequently, an NIR fluorescent probe was created by Tang et al. for the monitoring and identification of O_2_˙^−^ in relation to drug-induced liver injury in live cells and animals (Figure 8C) [65]. In the presence of O_2_˙^−^, the trifluoro group deprotects, producing the fluorophore LW-OH, which boosts the NIRF signal. This probe has a detection limit as low as 46.5 nM.

### 4.5. Fluorescent Probes for Peroxynitrite Detection (ONOO^−^)

**Figure 9:**
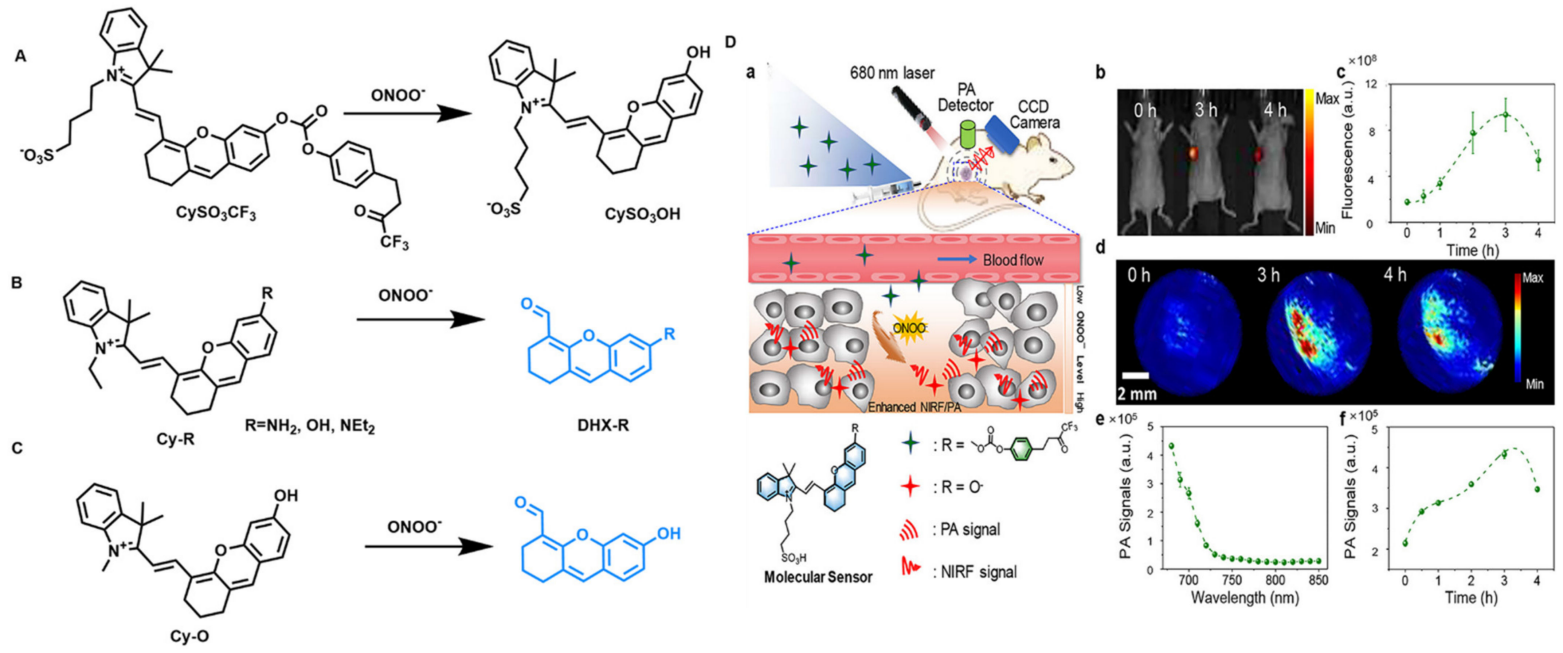
The fluorescence probe’s response mechanisms and chemical composition for ONOO^−^ for (**A**) CySO_3_CF_3_, (**B**) Cy-R, and (**C**) Cy-O. (**D**) In vivo NIRF and PA imaging of ONOO^−^ using CySO_3_CF_3_. (**a**) Illustration of the mechanism for NIRF and PA imaging of ONOO^−^ in tumor using CySO_3_CF_3_. CySO_3_CF_3_ first accumulates into tumor and then is activated by ONOO^−^ through cascade oxidation-elimination reactions, eventually resulting in enhanced NIRF/PA signals. (**b**) Real-time fluorescence imaging of tumor bearing mouse after systemic administration of CySO_3_CF_3_ (50 μM in 100 μL saline) at 0, 3, and 4 h. (**c**) Fluorescence intensity in tumor area as a function of postinjection time for CySO_3_CF_3_ intravenous injected mice. (**d**) Representative PA maximum imaging projection (MIP) of tumor from a systemic administration of a living mouse at 0, 3, and 4 h postinjection of CySO_3_CF_3_ (50 μM in 100 μL saline). (**e**) In vivo real-time PA spectra extracted from the tumors in living mice after systemic administration of CySO_3_CF_3_ via intravenous injection for 3 h. (**f**) PA intensity in tumor area as a function of postinjection time for CySO_3_CF_3_ intravenous injection. Reproduced with permission from Ref. [66]. Copyright (2018) American Chemical Society.

ONOO^−^ is a potent biological oxidant that can be generated in mitochondria by superoxide anion radicals and nitric oxide (NO), without enzymatic catalysis (O_2_^·−^). On the one hand, the oxidative, nucleophilic, and nitrifying activity of ONOO^−^ also plays an active role in many cellular signaling pathways. On the other hand, it damages a variety of biomolecules, including lipids, proteins, nucleic acids, and transition metal enzyme centers in an irreversible manner, resulting in a number of illnesses. There is increasing evidence that abnormal ONOO^−^ levels play a crucial causative role in certain diseases, such as neurodegenerative diseases, hepatotoxicity, diabetes, autoimmune diseases, inflammation, and cancer. Pu et al. produced a dual-modal fluorescent probe, CySO_3_CF_3_, for ONOO^−^ detection (Figure 9A) [66]. This probe modifies the trifluoromethyl ketone moiety into hemicyanine dyes as the ONOO^−^ responsive group. When ONOO^−^ exists, the fluorescence signal increases rapidly, so as to achieve the purpose of detecting ONOO^−^. CySO_3_CF_3_ displayed good biocompatibility and low cytotoxicity. The probe was then effectively used to identify ONOO^−^ in tumors using photoacoustic and fluorescence signals. Li et al. proposed a ratiometric fluorescent probe, Cy-R, which can improve the detection accuracy and effectively avoid the interference of certain external factors (Figure 9B) [67]. This probe offers a significant emission shift of roughly 250 nm, which is advantageous. The fluorescence signal at 710 nm decreased, while the fluorescence signal at 460 nm rapidly increased in the presence of ONOO^−^. Additionally, this probe offers a low 33 nM detection limit. In 2021, Zhang et al. also fabricated two similar fluorescent probes (Cy-O, Cy-N), and an encapsulated Cy-O through lipid A nanoprobe (NRF) was formed and used for in vivo and in vitro detection of ONOO^−^ (Figure 9C) [68]. NRF exhibits high sensitivity to ONOO^−^, a 1000-fold increase over other potential biological species, with outstanding selectivity for ONOO^−^. Nanoliposome encapsulation considerably increased the biocompatibility, while also improving the fluorescence performance. These findings unambiguously show that this probe is a candidate probe with excellent development potential and photostability for identifying the roles of ONOO^−^ in oxidative-stress-related activities under various physiological and pathological circumstances when diagnosing clinical tumors.

### 4.6. Fluorescent Probes for the Detection of H_2_S

H_2_S emits endogenous gases and has the aroma of rotting eggs. It appears to be engaged in a variety of physiological processes, controlling numerous critical physiological processes in the gastrointestinal, immunological, endocrine, neurological, and cardiovascular systems. Three methods can be used to create endogenous H_2_S from cysteine and homocysteine. The body’s homeostasis depends on the level of H_2_S; therefore, a deficiency can lead to significant illnesses, such as preeclampsia, Down syndrome, atherosclerosis, liver cirrhosis, and other conditions. The discovery of H_2_S biology and the treatment of associated disorders depend greatly on the development of sensitive molecular probes for the in vivo imaging of H_2_S.

**Figure 10:**
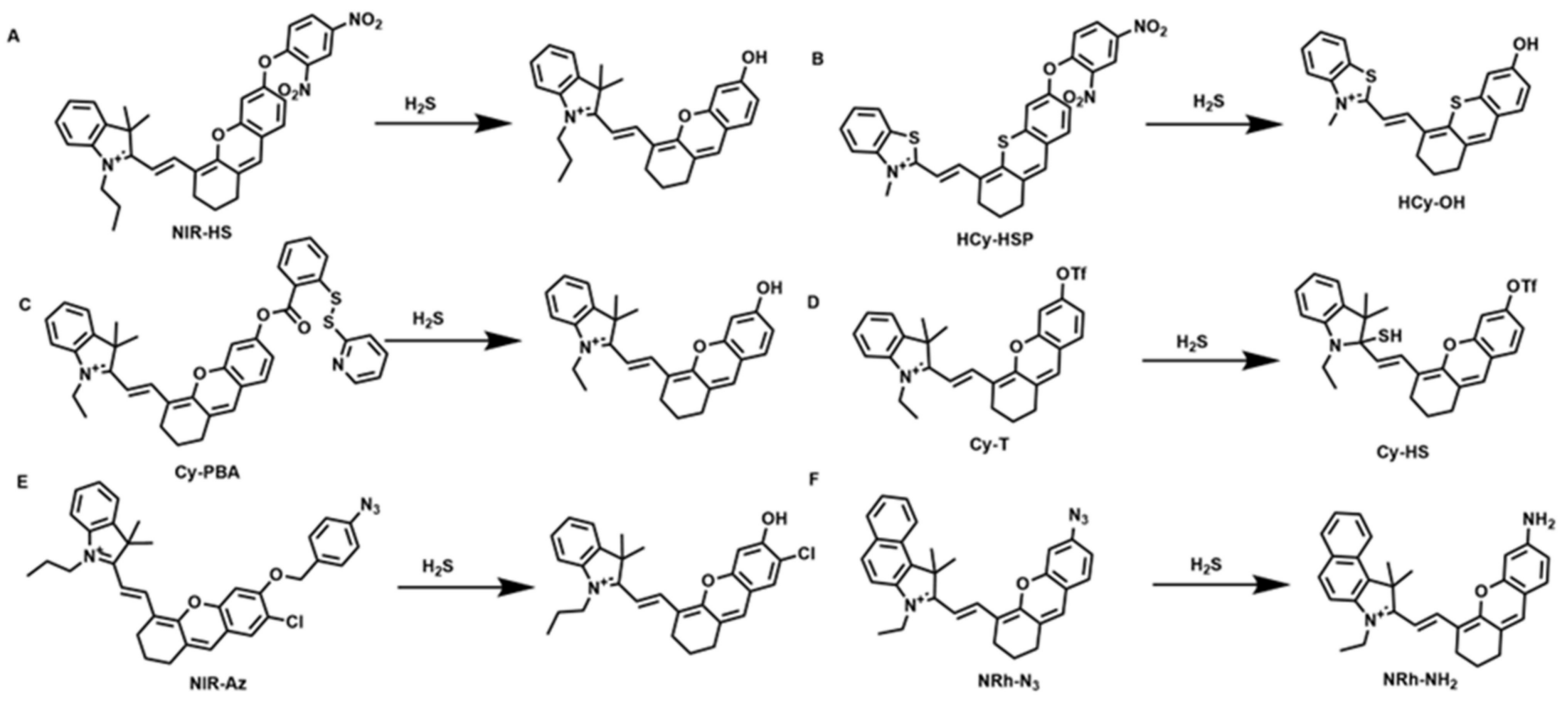
The response mechanisms and chemical structures (**A**) NIR-HS, (**B**) HCy-HSP, (**C**) Cy-PBA, (**D**) Cy-T, (**E**) NIR-Az and (**F**) NRh-N_3_ of fluorescent probes for H_2_S.

NIR-HS, a small-molecule fluorescent probe with the ether bond of dinitrobenzene serving as an H_2_S-responsive group, was first described by Zhang in 2016 (Figure 10A) [69]. The background signal of the probe was low, but the fluorescence intensity increased rapidly in the presence of Na_2_S. The detection limit was calculated to be 38 nM, and this probe also demonstrated a strong linear relationship with H_2_S. In addition to having excellent selectivity, NIR-HS has high sensitivity to H_2_S. This probe can identify the endogenous H_2_S produced in cells overexpressing cystathionine β synthase (CBS) and allows the fluorescence imaging of the endogenous H_2_S triggered by SNP in living cells. Exogenous and endogenous H_2_S were successfully seen using the probe in live mice. The probe NIR-HS has demonstrated potential as an important research tool for investigating the biological function of H_2_S. A novel NIR hemicyanine fluorescent probe called HCy-HSP was recently created and assembled by Liu et al. for the in situ imaging of H_2_S upregulation (Figure 10B) [70]. The excellent spectrum characteristics of the HCy-HSP probe include quick reaction times, high sensitivity, and outstanding H_2_S selectivity. The substitution of sulfur atoms can intentionally alter the wavelength to prevent the interference of the background fluorescence signal, which would allow for “on-and-off” H_2_S detection using the internal standard of HCy-HSP emission. The first in vivo and in vitro imaging of LPS-induced H_2_S buildup was made possible by HCy-HSP. This probe can be utilized as an indicator for the real-time evaluation of acute lung injury, since HCy-HSP can be used to observe the elevation of H_2_S levels during acute lung injury. In 2018, Zhang et al. designed and synthesized a new “turn-on” NIR fluorescent probe, Cy-PBA (Figure 10C) [71]. 2-(pyridin-2-yl-disulfanyl)-benzoic acid acts as a H_2_S-responsive group and can accurately distinguish it from other sulfides. Easy to manufacture, Cy-PBA has a quick response time of 1.5 min, good selectivity and sensitivity (LOD = 21 nM), and low cytotoxicity to H_2_S. Based on these advantages, Cy-PBA can be used to monitor H_2_S in living cells, animals, and tissues. Subsequently, Guo et al. reported a ratiometric fluorescent H_2_S-specific probe (Figure 10D) [72]. This novel probe exhibits outstanding sensing capabilities and can be modified to become a hemicyanine. First, CyT has excellent sensing properties for other biologically relevant anions and cations, as well as active sulfur; at the same time, it has a lower limit of detection (LOD) (7.33 nM) for H_2_S. Furthermore, CyT exhibits a fast response to H_2_S (rate constant of 1464 M^−1^ s^−1^) and a distinct color, ranging from dark to very pale blue, which can be observed with the naked eye. In addition, it could be used to successfully detect H_2_S in zebrafish and has negligible cytotoxicity to HeLa cells. With a self-immolative linker on the azide moiety and a phenolic dihydroxyanthracene generated from a cyanine dye, Yoon et al. designed and synthesized the NIR-Az (Figure 10E) NIR probe for the detection of hydrogen sulfide linkers incorporated between the fluorine groups [73]. This approach demonstrated selective fluorescence enhancement responses for H_2_S from different physiological species. The detection limit of the NIR-Az probe in PBS buffer solution was less than 0.26 μM, and it was highly selective for H_2_S among the 16 analytes examined, as well as other common reducing species. After NIR-Az treatment, the fluorescence was enhanced by more than 200 times, and the quantum yield after H_2_S treatment was 0.72. NIR-Az could both qualitatively and quantitatively detect hydrogen sulfide, utilizing time-dependent fluorescence processes. The observed range of endogenous hydrogen sulfide concentrations was covered by the obtained concentration linear relationship. The ability of NIR-Az to visualize hydrogen sulfide in living cells has been demonstrated, and this ability may be extended to assays involving biological fluids such as serum, blood, or homogenized tissue. NIR-Az exhibits a high turn-on response for the detection of hydrogen sulfide in living cells and mice. For the detection of H_2_S, both in vivo and in vitro, Liu et al. created an extremely sensitive and selective NIR fluorescent chemometer, NRh-N_3_ (Figure 10F) [74]. The NRh-N_3_ probe has good biosafety, stability, and detection limits. The probe’s ability to track the H_2_S release from medication interactions in living cells and small animals is significant. These discoveries have significant ramifications, in addition to offering a potential platform for the detection of H_2_S. This probe also offers a useful technique for H_2_S monitoring in clinical medicine, which is important for the development and release of medications.

### 4.7. Fluorescent Probes for Hydrogen Polysulfide Detection (H_2_S_n_)

**Figure 11:**
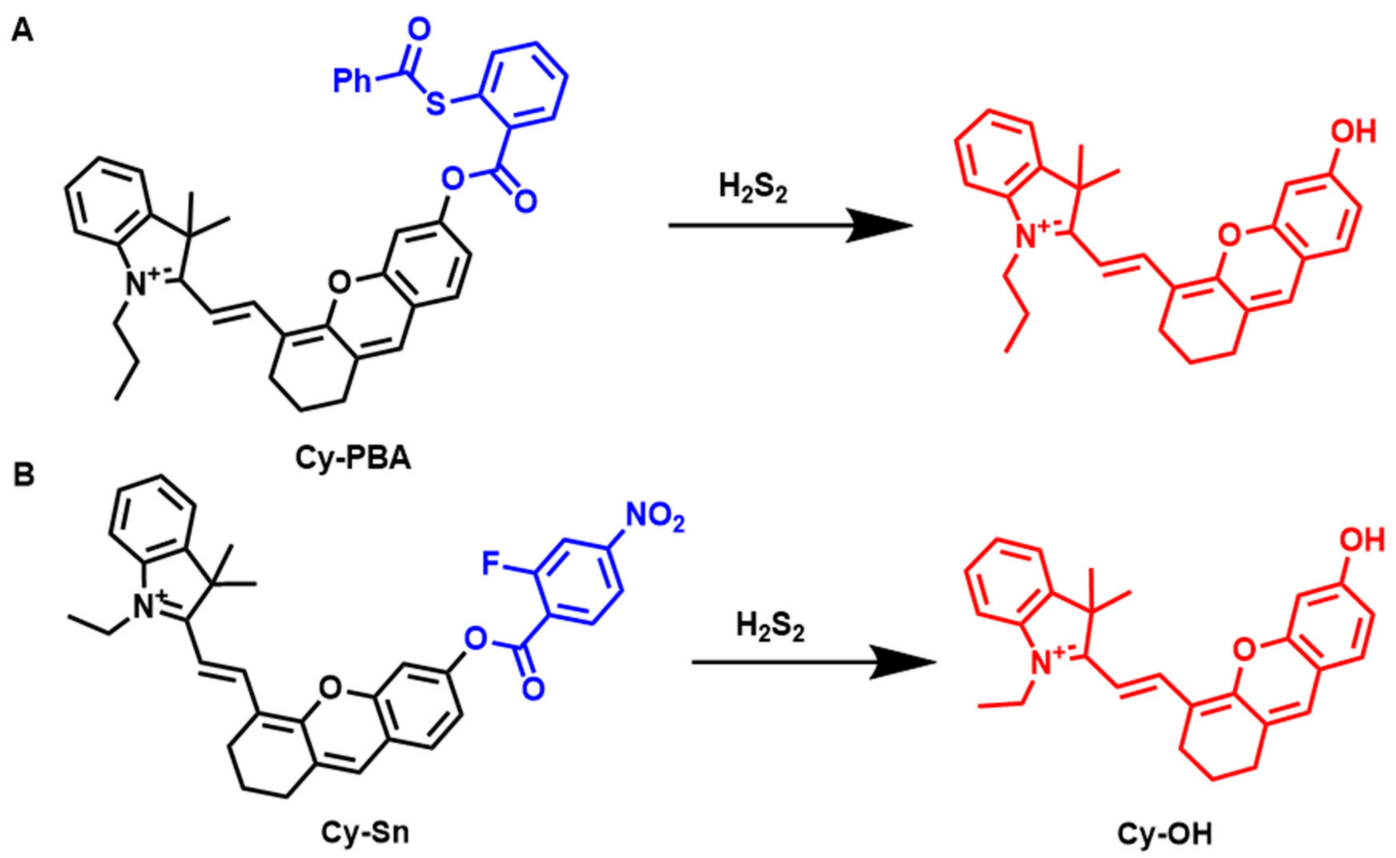
The response mechanisms and chemical structures (**A**) Cy-PBA and (**B**) Cy-Sn of fluorescent probes for H_2_S_2_.

Recent research suggests that H_2_S_n_ may be a novel signaling molecule and may be responsible for the biological functions attributed to H_2_S. For instance, H_2_S_n_ induces astrocyte Ca^2+^ influx several hundred times more effectively than H_2_S. Furthermore, many results have shown that H_2_S_n_ is crucial in biological systems, since it not only plays a crucial role in the S-sulfuration of numerous proteins, but also activates transcription factors, ion channels, and tumor suppressors. By coupling phenyl 2-(benzoylthio) benzoate with a hemicyanine backbone, Ma et al. created a novel NIR fluorescent probe with high sensitivity and selectivity, as well as a quick response to H_2_S_n_. (Figure 11A) [75]. It is important to note that this probe can be utilized to observe changes in H_2_S_n_ in living cells and animals. The detection limit was determined to be 35 nM. The extraordinary qualities of this probe make it an excellent candidate for use in different biological systems. Cy-Sn, a near-infrared probe created by Peng et al., responds only to H_2_S_n_ and has a detection range as low as 2.2 × 10^−8^ M, as well as a low cytotoxicity to biological systems (Figure 11B) [76]. Cy-Sn has been used to visualize endogenously generated H_2_S_n_ during inflammation and in situ anti-inflammatory processes via the CSE enzymatic pathway. Cy-Sn can identify H_2_S_n_ in living organisms, according to mouse H_2_S_n_ imaging investigations. Additionally, sodium polysulfides in stock solutions can be measured using Cy-Sn. As a result, this novel probe is capable of identifying H_2_S_n_ both in vitro and in vivo.

### 4.8. Fluorescent Probes for the Detection of Cysteine (Cys)

The most prevalent biothiols in the body are cysteine (Cys), glutathione (GSH), and homocysteine (Hcy). These sulfur-containing amino acids are crucial for preserving the redox balance in living organisms. In its functional state, Cys interacts with proteins and enzymes and is intimately linked to a number of illnesses, including neurotoxicity, liver damage, and hair coloring, as shown by aberrant variations in concentration. By identifying biological thiols in vivo, it is possible to indirectly monitor the concentration of proteins and enzymes that contain sulfur; this technique has significant potential for use in clinical diagnostics. Therefore, it is crucial to create an in vivo detection method for thiols that is precise, focused, and easy to use.

**Figure 12:**
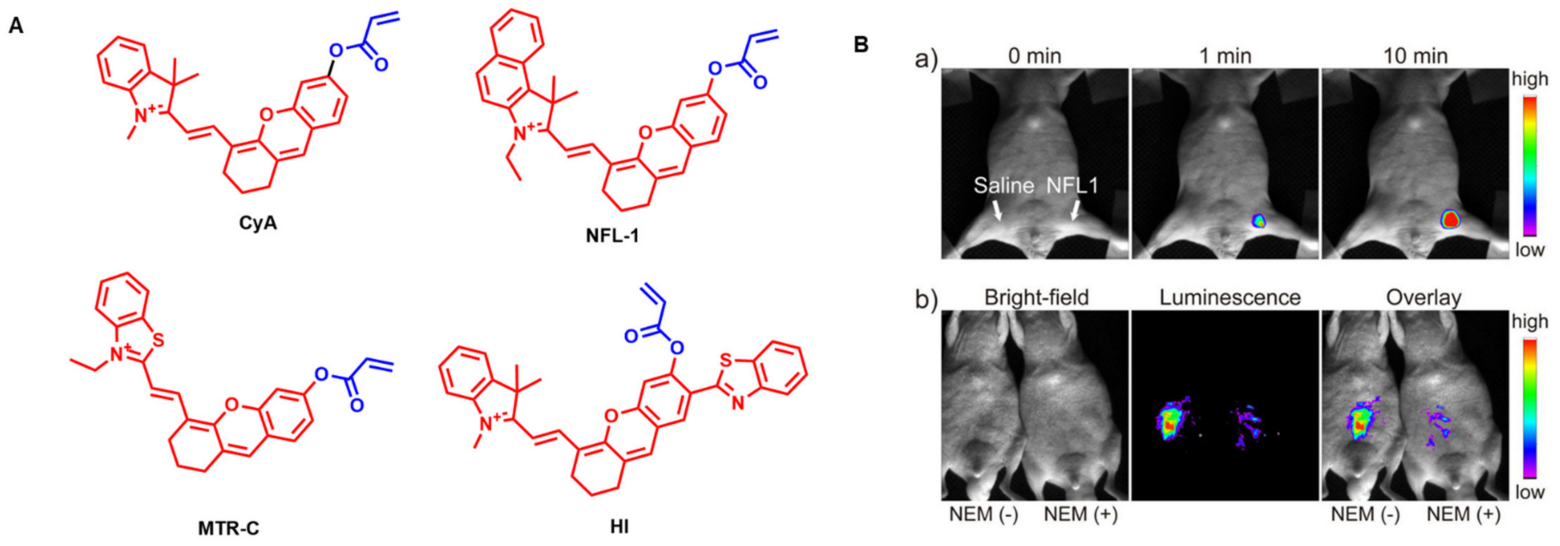
(**A**) Chemical structures of CyA, NFL-1, MTR-C, and HI; (**B**) In vivo NIR images of living mice via NFL1. (**a**) In vivo NIR images of living mice treated with 0.1 mL of saline (left side) or NFL1 (10 μM, right side) solution at different times after subcutaneous injection. (**b**) In vivo NIR images of living mice. The left mice were injected with probe NFL1 (10 μM, 0.2 mL) only for 10 min. Reproduced with permission from Ref. [78]. Copyright (2015) American Chemical Society.

**Figure 13:**
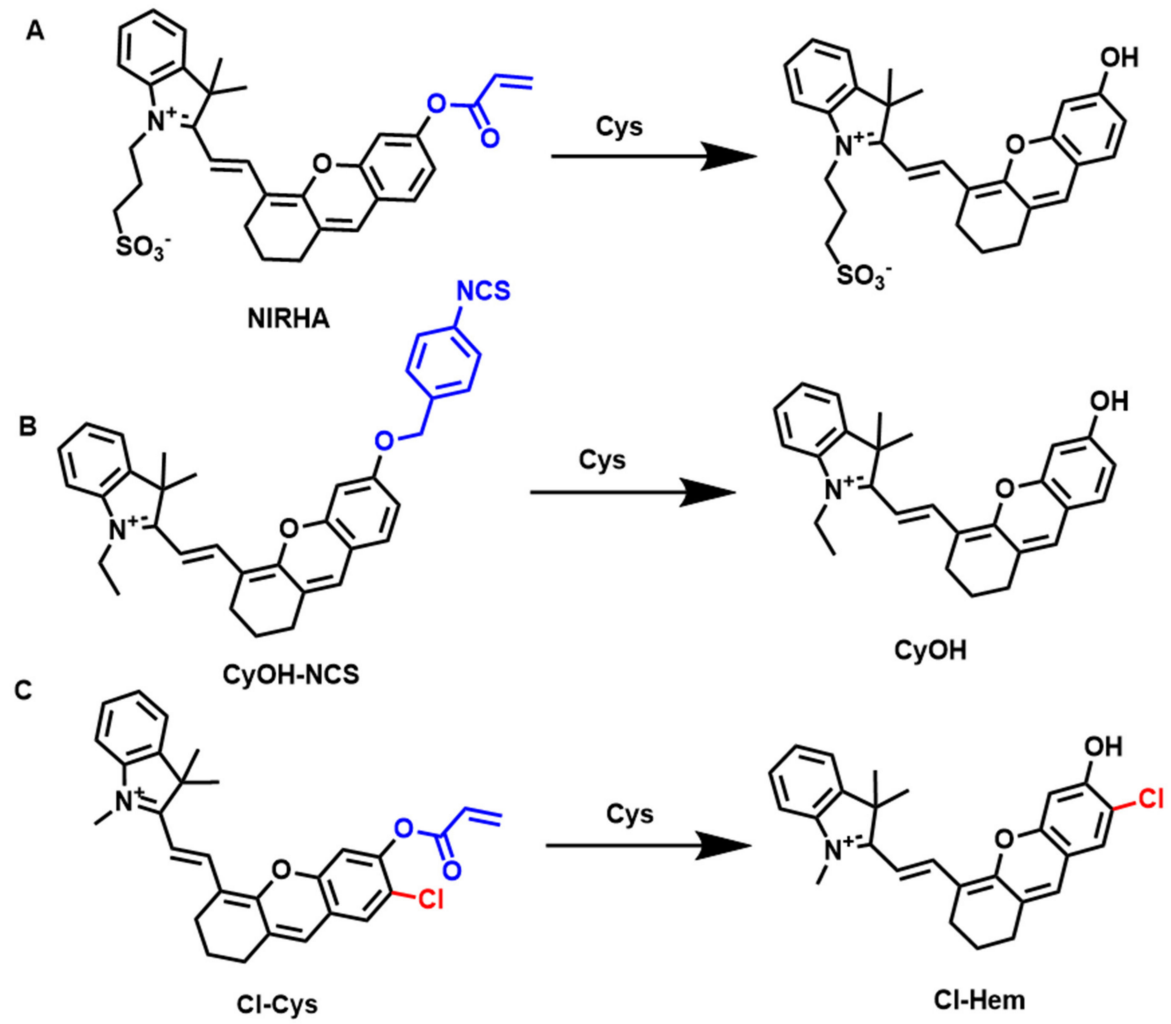
The response mechanisms and chemical structures (**A**) NIRHA, (**B**) CyOH-NCS, and (**C**) Cl-Cys of fluorescent probes for Cys.

In 2015, Professor Zhang’s team designed a hemicyanine NIR probe CyA with an acrylate group as the Cys recognition group, which improved the selectivity of Cys [77]. Here, Cys reacts with a,b-unsaturated carbonyl groups and selectively reacts with cysteine via intramolecular cyclization. The development of this mechanism greatly stimulated subsequent research, which has been important in cysteine probe design. Subsequently, some similar fluorescent probes were developed (Figure 12) [78,79,80]. Li et al. were responsible for the design and synthesis of a highly water-soluble near-infrared probe (NIRHA) (Figure 13A) [81]. The probe displayed excellent selectivity for Cys, which can specifically identify acrylate moieties and release NIR fluorescence signals. Mass spectrometry was used to confirm that the probe’s mode of action was accurate. The probe has a high NIRHA water solubility and can be used with practically all aqueous solutions, due to the addition of sulfonate. Owing to NIRHA’s low cytotoxicity, this probe also proved to be quick in a Cys assay (15 min), and it has successfully been used in cells and in vivo. Zhao et al. constructed a functional isothiocyanate self-immolation trigger-based NIR probe, CyOH-NCS, for specific detection of Cys in vitro and in vivo for the first time (Figure 13B) [82]. They integrated a cascade of nucleophilic cyclization and self-immolative degradation into NIR hemicyanine dyes. CyOH-NCS is more selective for Cys than other biothiols. Cys is perceived based on a self-immolation strategy. CyOH-NCS probes have been successfully used to image Cys in live cells, zebrafish, and mice. Furthermore, this probe also validates that isothiocyanate-functional molecules can act as efficient H_2_S donors, thus providing a promising prospect for the development of bifunctional probes for fluorescence imaging and pathophysiology applications. In 2022, Kolemen et al. designed a synthesis furnace for the detection of cysteine fluorescent probes (Cl-Cys) (Figure 13C) [83]. These probes do not suffer from the drawbacks of poor photostability, high toxicity, high energy absorption signal, and low water solubility. When reacting with Cys, Cl-Cys has an amazing turn-on response, in terms of its NIR emission signal, activating its singlet oxygen generation, and photothermal conversion potential. The chlorinated hemicyanine nuclei can be used as dual phototherapeutics at high doses, without carrying heavy atoms. Furthermore, Cl-Cys only activates an intense fluorescence in cancer cells, allowing for tumor cell localization and imaging, as well as highly targeted image-guided phototherapy. Cl-Cys is the first therapeutic drug that can be triggered by Cys, in addition to being the first instance of Cys-responsive phototherapy. It is important to emphasize that dual phototherapy is still infrequently induced by activity-based cancer-cell-selective photosensitizers.

### 4.9. Fluorescent Probes for the Detection of GSH

In mammalian cells, GSH is the most prevalent non-protein thiol and is essential for both physiological and pathological processes, such as cell metabolism, proliferation, differentiation, and death. In general, the intracellular GSH concentration is approximately 0.5–10 mM. In cancer cells, its content is more abundant, especially in liver cancer cells. GSH is very important in maintaining the homeostasis of redox in cells, and it can effectively resist the invasion of foreign substances, such as ROS, RNS, etc. When its levels are abnormal it is linked to numerous human disorders, including ageing, cancer, and cardiovascular, immunological, and neurological conditions. Therefore, the creation of efficient techniques capable of observing and photographing the dynamic variations in GSH in living cells in real time is crucial for obtaining pathophysiological insights and diagnosing associated disorders.

**Figure 14:**
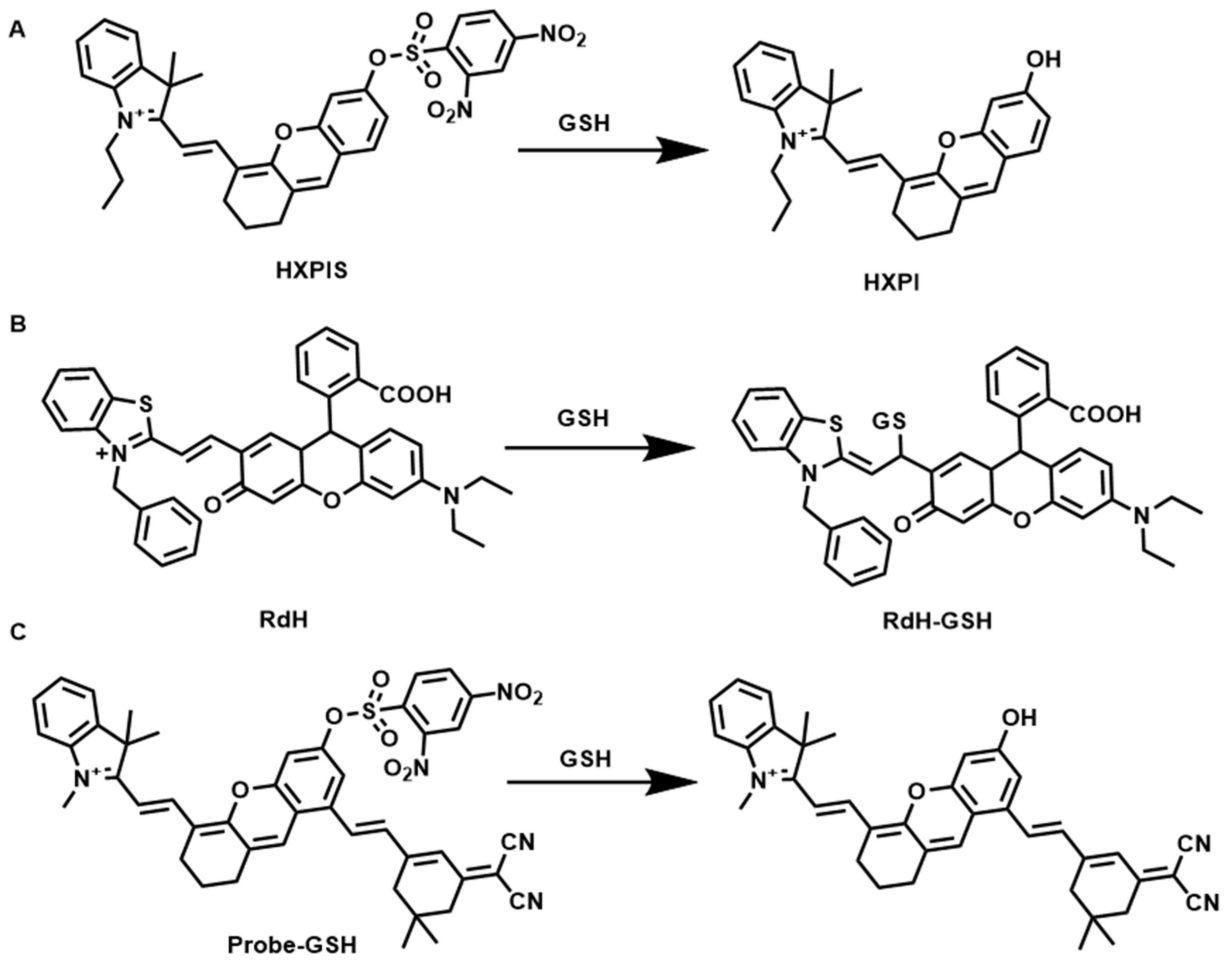
The response mechanisms and chemical structures (**A**) HXPIS, (**B**) RdH, and (**C**) Probe-GSH of fluorescent probes for GSH.

By incorporating the 2,4-dinitrobenzenesulfonyl group as the recognized functional group of GSH into a hemicyanine fluorophore, Yu et al. created a new NIR probe, HXPIS (Figure 14A) [84]. The probes only showed distinct fluorescent signals in the presence of biothiols. HXPIS showed excellent sensitivity to GSH, with an LOD as low as 10.7 nM. Furthermore, HXPIS showed low cytotoxicity and good biocompatibility. This probe can not only be used for the detection of GSH in cells but also to test the biothiol concentration in bacteria. As the dissociation of the E. coli cell wall proceeds, the amount of clear red fluorescent signal tends to increase. HXPIS has also been used for the fluorescence imaging of rat liver tissue sites. In the same year, Guo et al. proposed a new amaranth–hemicyanine dye, RdH (Figure 14B) [85]. Here, probes containing activated double bonds can rapidly, selectively, and reversibly react with GSH, resulting in the generation of proportional fluorescent signals. Furthermore, the fluorescence imaging of variations in GSH content in living cells using this probe has proven successful. Recently, Li et al. modified conventional hemicyanine dyes to create a fluorescent probe (probe-GSH) that can be employed for the dual-modal detection of GSH (Figure 14C) [86]. They suggested using a vinyl bridge to graft dicyanoisophorone onto the hemicyanine dye’s backbone, in order to enlarge its conjugated structure. This would accomplish the goal of extending the absorbance/emission wavelength. Dicyanoisophorone and indole iodide are anthracene moieties that act as electron-withdrawing groups (acceptors) in this structure. Vinyl bridges are used as π bridges, and hydroxyl groups are used as analyte recognition moieties as well as electron donors. The 2,4-dinitrobenzenesulfonyl ester group (donor) serves as the responsive moiety for GSH. The activated probe-GSH exhibited an eightfold enhancement in the photoacoustic signal/10-fold fluorescence intensity. More notably, the probe-GSH was effectively applied to the fluorescence and photoacoustic dual-mode imaging of 4T1 tumor-bearing mice.

### 4.10. Carbon Monoxide Detection Using Fluorescent Probes (CO)

CO is a colorless and odorless gas that is highly poisonous. However, new research indicates that carbon monoxide, similarly to nitric oxide (NO) and hydrogen sulfide (H_2_S), is a gas transmission factor in the human body and plays an important role in a variety of physiological and pathological processes. It not only participates in vasodilation, neurotransmission, and anti-inflammatory processes, but its metabolic abnormalities are also related to many diseases, including Alzheimer’s disease, inflammation, cancer, and neurological diseases. As a result, CO has become a popular research topic in recent years. Carbon monoxide is thought to decrease the activity of cytochrome c oxidase, by competing with oxygen, thus damaging the mitochondrial membrane potential and, ultimately, leading to cell death. However, mitochondrial respiration is related to CO concentration. As it has many functions that we do not understand very well, it is meaningful to determine and monitor the level of carbon monoxide in organisms.

**Figure 15:**
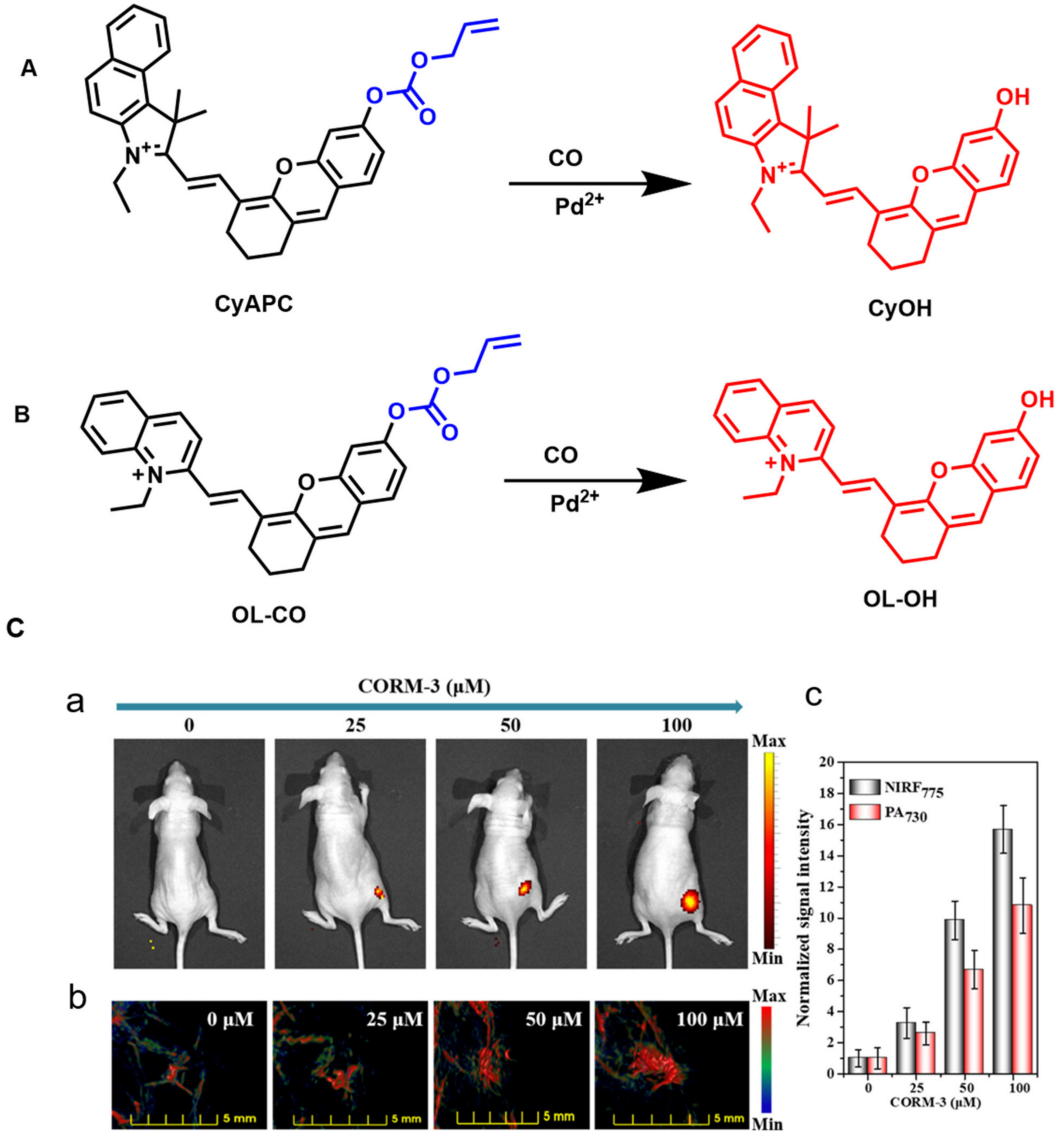
The response mechanisms and chemical structures (**A**) CyAPC and (**B**) OL-CO of fluorescent probes for CO. (**C**) In vivo imaging of CO in living mice. (**a**) Fluorescent imaging and (**b**) PA730 imaging of living mice treated with the probe system (QL-CO + PdCl_2_, 20 μM each) and various concentrations of CORM-3 (0, 25, 50, and 100 μM) in mice. (**c**) Normalized NIRF775 and PA730 signal intensity values from (**a**,**b**). Reproduced with permission from Ref. [88]. Copyright (2022) American Chemical Society.

Li et al. described a hemicyanine dye-based NIR fluorescence probe for detecting CO in mitochondria (Figure 15A) [87]. CyAPC is a positively-charged molecule that can clump together near cell mitochondria. CO and Pd^2+^ caused allyl formate group cleavage on the probe, resulting in an increased fluorescence signal. The NIR fluorescence probe was used to track changes in external and endogenous CO levels in a variety of biological samples (such as cells, tissues, and in vivo samples). In 2022, Lin et al. developed a new dye support, QL-OH, by optimizing the classical hemicyanine dyes (Figure 15B) [88]. Based on this improved dye, a dual-mode probe, QL-CO, for CO detection based on fluorescence and photoacoustic imaging was devised and produced. In the presence of CO, QL-CO showed a rapid and sensitive double activation reaction to CO. QL-CO could not only used for the non-invasive and sensitive display of CO levels in deep inflammatory lesions in vivo, but was also successfully used for the first time for in vivo inflammation diagnosis and the anti-inflammatory drug efficacy evaluation of mice through dual-mode imaging technology. In addition, the QL-CO probe can accurately locate deep inflammatory lesions (≈1 cm) in mice, and obtain spatially and deeply resolved three-dimensional photoacoustic diagnostic pictures. Deep tissue diagnostic imaging of CO in vivo using the new dual-mode QL-CO probe has considerable potential.

### 4.11. Fluorescent Probes for the Detection of Hydrazine (N_2_H_4_)

N_2_H_4_ is an important chemical reagent and is widely used in corrosion storage agents, catalysts, pharmaceuticals, and pharmaceutical intermediates. However, excessive exposure to N_2_H_4_ can seriously harm human health; for example, it can cause hepatotoxicity, neurotoxicity, and mutagenicity. Although there is no endogenous N_2_H_4_, it is easily absorbed through the skin, the mouth, or by respiration, thus causing harm to human health. Therefore, highly sensitive and selective micro N_2_H_4_ detection methods, especially fluorescent probes, are of great significance for the in vivo and in vitro detection of N_2_H_4_.

**Figure 16:**
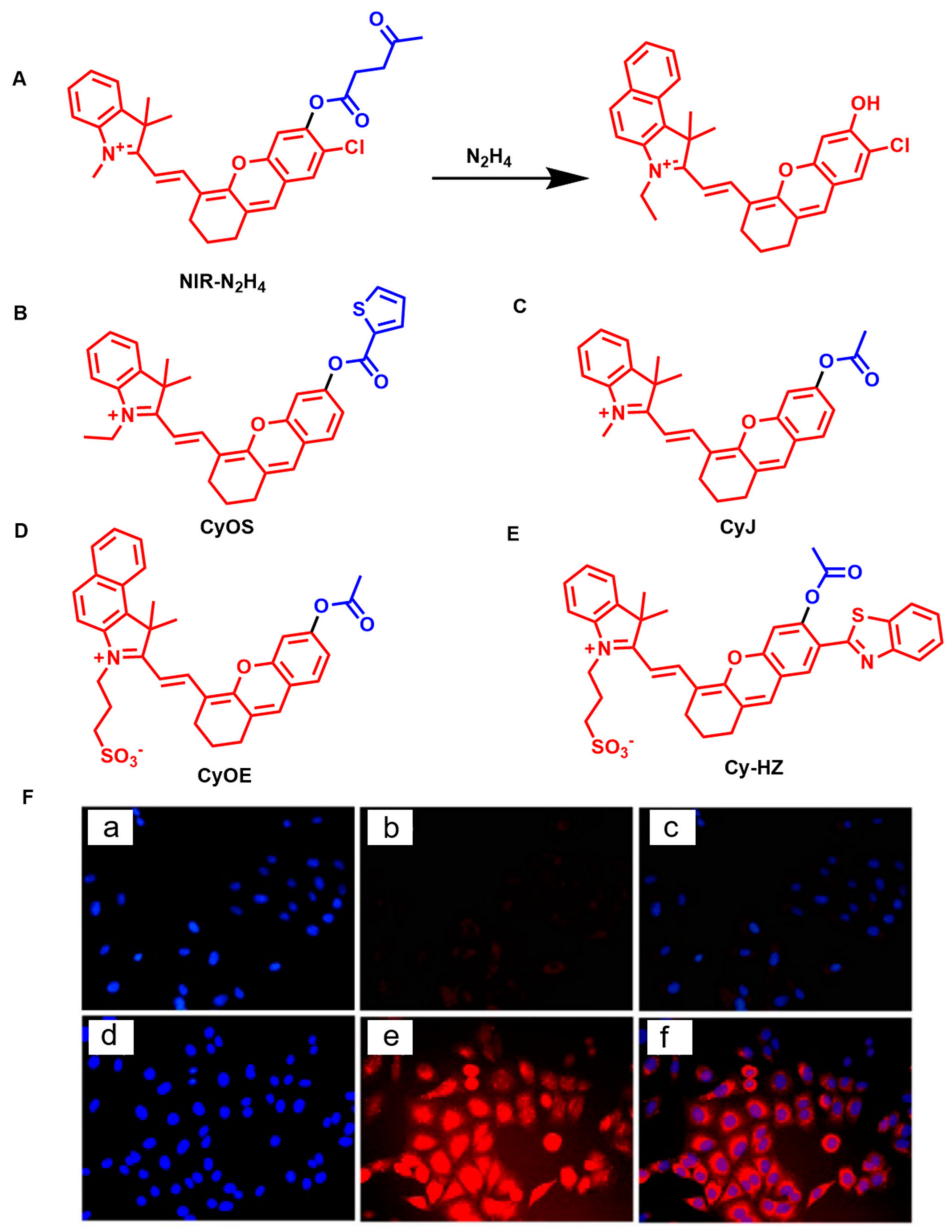
The response mechanisms and chemical structures (**A**–**E**) of fluorescent probes for N_2_H_4_. (**F**) Confocal microscope images of CyJ in HeLa cells. (**a**,**d**) Fluorescence images from DAPI; (**b**,**e**) Fluorescence images from CyJ; and (**c**,**f**) Merge. Reproduced with permission from Ref. [91]. Copyright (2015) American Chemical Society.

In 2013, Yuan et al. described the compound NIR-N_2_H_4_ as a new candidate to detect N_2_H_4_ in serum and cells. The levulinic acid group was introduced as the reaction group of N_2_H_4_ (Figure 16A) [89]. In the presence of N_2_H_4_, it mediates deprotection and causes a change in the fluorescence signal. The probe not only has a significantly high sensitivity and selectivity to N_2_H_4_ in the NIR region, but also performs well at healthy pH levels. Additionally, NIR-N_2_H_4_ can be utilized to image N_2_H_4_ in living cells and to measure N_2_H_4_ in serum samples. Li et al. doped the 2-thiophene carbonyl part into the hemicyanine fluorescent group to synthesize a new probe (CyOS) (Figure 16B) [90]. The probe has high selectivity for N_2_H_4_ compared with various amino acids and common ions. The fluorescence enhancement at 701 nm increases dramatically in the presence of N_2_H_4_, and the detection limit can drop as low as 0.78 ppb. This is the first visualization of N_2_H_4_ in deep living tissue.

Zhang et al. reported an NIR fluorescence probe, CyJ, for the detection of N_2_H_4_ (Figure 16C) [91]. The CyJ probe contains acetyl (as the recognition part) and a hemicyanine skeleton (as the fluorescence emission part). This new probe is not only easy to prepare but also has excellent sensing performance. Most notably, CyJ has a low detection limit (LOD) and is extremely selective for N_2_H_4_ when compared with a variety of anions, cations, and other amino compounds (5.4 ppb as a fluorescence sensor, 6.1 ppb as a UV sensor). Additionally, CyJ has low cytotoxicity and good cell membrane permeability. Additionally, it was possible, for the first time, to see N_2_H_4_ in the liver, lung, kidney, heart, and spleen of living mice using CyJ. In 2022, Hu et al. also reported a new near-infrared fluorescence probe, CyOE, based on the acetyl-containing hemicyanine dye as the recognition site (Figure 16D) [92]. The CyOE probe has high selectivity and sensitivity to N_2_H_4_ (LOD = 82 nM). In the design of the probe, the introduction of sulfonate increased the water solubility of the probe, providing a good basis for the detection of N_2_H_4_, while making biological considerations for the water present in the system. CyOE may successfully be used to visualize N_2_H_4_ in cells, in addition to being employed for the quick colorimetric and gas phase detection of N_2_H_4_ in aqueous solutions and actual water samples. A similar fluorescent probe was created by Ran et al. (Cy-HZ), which can detect N_2_H_4_ in mitochondria (Figure 16E) [93]. The probe has good biocompatibility and can visualize N_2_H_4_ and monitor the distribution and metabolism of isoniazid.

## 5. Conclusions and Outlook

In this study, we have outlined the advancements in small molecule fluorescent probes based on hemicyanine fluorophores, as well as their use in the detection and treatment of various disorders. In general, the fluorescent chromophore’s functional component (carboxyl, hydroxyl, or amino) can be altered, and a matching small-molecule fluorescent probe can then be constructed using the “protection and deprotection” technique. On the other hand, recovering or analyzing hemicyanine fluorophores offers an alternate framework for the development of fluorescent probes that are triggered and emit NIR light, which is vital to be observed in depth. These probes are crucial for the diagnosis of conditions such as arthritis, liver cancer, and other conditions of a similar nature, and the skeletons have also advanced, although there are still certain issues and difficulties. First, the majority of fluorescence probes have been developed to only detect and visualize a single biomarker in biological systems. A single physiological activity or metabolic process that is closely related to several diseases may involve multiple biomarkers. As a result, the identification of a single biomarker cannot provide sufficient information about a disease or fulfill the criteria for a precise diagnosis. Second, active fluorescence probes of the hemicyanine fluorophore have a very narrow light transmission range, mostly in the NIR-I. We can resolve this issue by making a modified NIR-II fluorescence probe; however, there are still some difficulties. Additionally, photoacoustic imaging, which generates ultrasonic signals using near-infrared light as the excitation source, has the potential to penetrate to centimeter-level depths. The signal-to-noise ratio of fluorescence detection in living cells and in vivo is decreased as a result of the comparatively poor fluorescence quantum yield of hemicyanine dyes and aggregation-induced quenching at high concentrations. This issue can be successfully avoided by incorporating the design notion of aggregation-induced emission. This review is intended to encourage the further use of hemicyanine dyes in chemical biology and medicine, by offering relevant information for their design, sensing, and biological application.

## Data Availability

Not applicable.
